# Supplementary material for: Relation between occupants’ health problems, demographic and indoor environment subjective evaluations: A cross-sectional questionnaire survey study in Java Island, Indonesia
Source: PLoS One. 2021 Jul 9;16(7):e0254460. doi: 10.1371/journal.pone.0254460 (PMC8270411; doi:10.1371/journal.pone.0254460)
Supplement: S1 Appendix — (PDF) [file pone.0254460.s001.pdf]

Appendix S1

Health Related to Indoor Environment Survey

**A. RESPONDENT DATA** *please fill it and choose the most appropriate one*
1. Age : .... 5. Estimated monthly income …………<br>2. Sex : Male / Female 6. Number of room in house…..<br>3. City/District type: .... House type : 1-storey / 2-storey / Apartment<br>4. Daily period of staying home :…….. Hours. From……to……

B. HEALTH PROBLEM RECORDS

1. Please check (√) the most appropriate option/s

Please give the most suitable health symptoms you have suffered in the last 3 months

Health Issue	Yes	No		Health Issue	Yes	No	
**1. Respiratory**				**2. Kidney & Urinary**			
a. Sinusitis				a. Difficulty urinating			
b. Bronchitis				b. Inflammation			
c. Cough				c. Prostate Inflammation			
d. TBC				d. Urolithiasis			
e. Pneumonia							
f. Asthma				**3. Neural**			
g. Dyspnea				a. Meningitis			
				b. Concussion			
**4. Digestive tract**				c. Polio			
a. Typhoid				d. Epilepsy			
b. Puke				e. Stroke			
c. Constipation				f. Migraine			
d. Ulcer							
e. Icterus				**5. Gland Symptoms**			
f. Cholelithiasis				a. Thyroid			
g. Hemorrhoids							
h. Diarrhea				**6. Skin Symptoms**			
i. Odynophagia				a. Chicken Pox			
				b. Tinea Versicolor			
**7. Other Symptoms**				c. Eczema			
a. Food Allergy							
b. Always Thirsty				**8. Heart and Blood**			
c. Medicine Allergy				a. Tachycardia			
d. Headache				b. Chest Pain			
e. Tetanus				c. Hypotension			
f. Fainting				d. Hypertension			
g. Tiredness							
h. Hearing disorder				**9. Lifestyle**			comments
i. Rheumatism				a. Smoking, if "yes" how many cigarettes per day			
j. Cancer				b. Drink Alcohol, if "yes" how many glasses per day			
k. Fidgeting				c. In medical treatment			
l. Damp skin				d. Frequently eat sweet and fatty food			
m. Muscle Cramps				e. Have family member who got symptoms, if "yes" what is the symptom)			
n. Measles				f. Don't have routine exercise			
o. Malaria				g. Frequently stay up late night			
p. Diabetes				h. Keep house unclean			
q. Insomnia							

C. DAILY INDOOR ENVIRONMENT CONDITIONS

a. How often did you get exposed to hot indoor air temperature? **No / Less frequent / A few days per month / A few days per week / Every day**

b. How often did you feel difficulty to sleep due to thermal discomfort during sleeping time? **No / Less frequent / A few days per month / A few days per week / Every**

c. While you are staying in the house, how do you feel about indoor air quality? **Very good / Good / Slightly poor / Poor**

d. If you answered (c) slightly poor or poor, in our opinion, what is its sources? **Smoke / Odor / Dust / Fungi /Other_____**

e. In general, what do you feel your indoor thermal environment? **Hot / Warm / Slightly warm / Neutral / Slightly cool / Cool / Cold**

f. In general, what would you say the condition of your indoor environment? **Very satisfied / Satisfied / Slightly dissatisfied / Dissatisfied**
