# Supplementary material for: Relation between occupants’ health problems, demographic and indoor environment subjective evaluations: A cross-sectional questionnaire survey study in Java Island, Indonesia
Source: PLoS One. 2021 Jul 9;16(7):e0254460. doi: 10.1371/journal.pone.0254460 (PMC8270411; doi:10.1371/journal.pone.0254460)
Supplement: Attachment — Submitted filename: Response to Reviewers.docx [file pone.0254460.s003.docx]

**Dear Reviewers,**

I, along with my co-authors, would like to express our appreciation to your comments on our paper titled “Association between the prevalence of occupants’ health problems to their demographic and subjective indoor environment quality: A cross-sectional questionnaire survey study in Java Island, Indonesia” (PONE-D-21-09960). The comments have helped us improve the manuscript. According to your comments and suggestions, we have revised the manuscript as tabulated as follows.

Reply for Reviewer #1

| # | Reviewer’s comments | Response |
| --- | --- | --- |
| R1-#1 | This sentence in the abstract 'Additionally, perceived indoor environment quality could influence health problems by mediating thermal discomfort experiences.', mediating should be changed with more suitable word. | According to your suggestion, we modified the sentence as: ‘Additionally, perceived indoor environment quality, which is possible to cause thermal discomfort experience, indirectly affect health problems’ (Line 29) |
| R1-#2 | Line 99: It should be susceptible. | We changed the word as your suggested. |
| R1-#3 | Line 182: People who expressed satisfaction were those who struggled to sleep or suffered indoor hear is in conflict. Please correct this sentence. | We corrected the sentence as: ‘Furthermore, based on respondents’ subjective evaluations, those who indicated that they were “very satisfied” with indoor environment conditions, generally show a small fraction of having difficulties to sleep in nighttime and suffered hot indoor air exposure.’ |
| R1-#4 | Line 188: Spelling of emphasized. | We changed the word as your suggested. |
| R1-#5 | Line 197: Indoor and outdoor air quality of slums in Chile were worse than urban areas. How does this correlate to rural areas? Slums are entirely different than rural areas. Please clarify or remove this statement. | We decided to remove this statement as you suggested. |
| R1-#6 | In respiratory health conditions, authors mention 'blown' as a condition. What is it? I could only find on google that this term is used to emphasize disease severity. if it is a disease itself, please clarify in text or table. | According to your suggestion, we replaced it with ‘Dyspnea’. It is the medical term for shortness of breath. |
| R1-#7 | Table 2: I guess authors meant 'Fidgeting and Fainting' instead of Fidgety and Fainted. Same in rest of text. Please cross-check which word suits where. | Thank you for your advice. We replaced ‘fidgety’ and ‘fainted’ with ‘fidgeting’ and ‘fainting’ in the entire manuscript. |
| R1-#8 | Do check if odds ratio is less than 1, it is negatively correlated instead of positive correlation | We re-checked it and modified the text in line 220 as: ‘An OR less than 1 implies a negative relationship and vice versa’. Additionally, we modified the statements to be clearer in line 235 as follow:  “a negative value of the estimated *β_j_* coefficients (OR<1) indicates a negative association between the prevalence of the health problem and the corresponding covariate. It means that respondents from a certain group of the corresponding covariate are less prone to the specific health problem compared to those in the reference category. Similarly, OR>1, which occurs for a positive value of *β_j_* estimates, indicates higher vulnerability of that specific group of the respondents to the health problem of interest than those of the reference group.” |
| R1-#9 | Authors should also mention or elaborate if there are some conditions that are genetic and may not be consequence of environmental conditions. In case of hypertension, it might be linked to both environment or genetics in a population. | Thank you very much for your insightful suggestion.  As you suggested, it has been known that the interaction between genetic and environmental factors play an important role in hypertension based on past various medical studies. Considering the limited number of questions and participants of our survey, we think it is difficult to discuss about “whether there are some conditions that are genetic and may not be consequence of environmental conditions”. Although we cannot fully respond your suggestion, we modified the manuscript to be  “With regard to hypertension, it has been long known that interactions of multiple genetic and environmental factors play a significant role, and identified several indices associated with prevalence of hypertension such as body mass index (Pazoki et al., 2018; Peltzer and Pengpid, 2018; Zanchetti, 2016). Because of the limitation of sample number and type of questions, the survey cannot draw insight related to these factors, we can confirm that the prevalence of hypertension tends to be significantly correlated with the age group older than 50 years since its odds ratio is less than one; the lowest of all the age groups. Although the sample number of our survey is not quite large, this tendency is consistent with the Indonesian Ministry of Health’s report, which confirmed that 41% of adults older than 60 years old exhibited the hypertension in 2012, which is higher than the value of 17.1% for the age group of less than 25 years old (Giena et al., 2018; Widjaja et al., 2013).” (Line 247).  References:  Giena, V.P., Thongpat, S., Nitirat, P., 2018. Predictors of health-promoting behaviour among older adults with hypertension in Indonesia. Int. J. Nurs. Sci. 5, 201–205. https://doi.org/10.1016/j.ijnss.2018.04.002  Pazoki, R., Dehghan, A., Evangelou, E., Warren, H., Gao, H., Caulfield, M., Elliott, P., Tzoulaki, I., 2018. Genetic predisposition to high blood pressure and lifestyle factors: Associations with midlife blood pressure levels and cardiovascular events. Circulation 137, 653–661. https://doi.org/10.1161/CIRCULATIONAHA.117.030898  Peltzer, K., Pengpid, S., 2018. The Prevalence and Social Determinants of Hypertension among Adults in Indonesia: A Cross-Sectional Population-Based National Survey. Int. J. Hypertens. 2018. https://doi.org/10.1155/2018/5610725  Widjaja, F.F., Santoso, L.A., Barus, N.R.V., Pradana, G.A., Estetika, C., 2013. Prehypertension and hypertension among young Indonesian adults at a primary health care in a rural area. Med. J. Indones. 22, 39–45. https://doi.org/10.13181/mji.v22i1.519  Zanchetti, A., 2016. Genetic and environmental factors in development of hypertension. J. Hypertens. 34, 2109–2110. https://doi.org/10.1097/HJH.0000000000001102 |

Reply for Reviewer #2

| # | Reviewer’s comments | Response |
| --- | --- | --- |
| R2-#1 | Understanding the basic relationship structure between the built environment, anthropometric factors, and economic conditions for a specific region is vital. Hence, this study intends to reveal the relationship between perceived indoor environments, experiences of indoor thermal discomfort, and occupant health conditions in Indonesia by means of a subjective questionnaire survey. Furthermore, the factors related to indoor environment quality (IEQ), economic levels, locations, and basic anthropometric information are also considered as potential explanatory variables for health condition. The manuscript is well written, and addresses are the research questions. Therefore, can be accepted in this current form. | Thank you for giving us beneficial comments for our present work. |

Reply for Reviewer #3

| # | Reviewer’s comments | Response |
| --- | --- | --- |
| R3-#1 | The paper results and their details of contained have original work, but the under the ethical issue, of avoiding displaying the photo of people whose get the information of data and get their approval first. In my opinion, the title needs more concise. The supporting information, in particular, the maps, it needs professional maps and clear. | Thank you very much for your constructive insights.  We modified the tittle more concise as you suggested.  The updated title: Relation between occupants’ health problems, demographic and indoor environment subjective evaluations: A cross-sectional questionnaire survey study in Java Island, Indonesia  Additionally, we have modified the supporting information. We deleted photograph of participants in the study because we unable to obtain the consent form the subject of the photograph.  . |
